# Supplementary material for: COZOID: contact zone identifier for visual analysis of protein-protein interactions
Source: BMC Bioinformatics. 2018 Apr 6;19:125. doi: 10.1186/s12859-018-2113-6 (PMC5889581; doi:10.1186/s12859-018-2113-6)
Supplement: Supplementary file 2 — Software build. Executable binary file of the software tool. (ZIP 118,784 kb) [file 12859_2018_2113_MOESM2_ESM.zip › cozoid/platform/docs/testng-6.5.1-javadoc.zip!/org/testng/IObjectFactory.html]

IObjectFactory (TestNG)


---


|  |  |  |
| --- | --- | --- |
| **Overview** \| **Package** \| **Class** \| **Tree** \| **Deprecated** \| **Index** \| **Help** | |  |
| **PREV CLASS**   **NEXT CLASS** | **FRAMES**    **NO FRAMES**     **All Classes** |  |
| SUMMARY: NESTED \| FIELD \| CONSTR \| METHOD | DETAIL: FIELD \| CONSTR \| METHOD |  |


---


## org.testng Interface IObjectFactory

**All Superinterfaces:**: ITestObjectFactory, java.io.Serializable

---

``` public interface IObjectFactory extends ITestObjectFactory ```

Factory used to create all test instances. This factory is passed the constructor
along with the parameters that TestNG calculated based on the environment
(@Parameters, etc...).

**Since:**
:   5.6

**Author:**
:   Hani Suleiman

**See Also:**: `IObjectFactory2`

---

| **Method Summary** | |
| --- | --- |
| `java.lang.Object` | `newInstance(java.lang.reflect.Constructor constructor, java.lang.Object... params)` |

**Method Detail**

### newInstance

```
java.lang.Object newInstance(java.lang.reflect.Constructor constructor,
                             java.lang.Object... params)
```


---


|  |  |  |
| --- | --- | --- |
| **Overview** \| **Package** \| **Class** \| **Tree** \| **Deprecated** \| **Index** \| **Help** | |  |
| **PREV CLASS**   **NEXT CLASS** | **FRAMES**    **NO FRAMES**     **All Classes** |  |
| SUMMARY: NESTED \| FIELD \| CONSTR \| METHOD | DETAIL: FIELD \| CONSTR \| METHOD |  |


---
